# A lymph node–targeted Amphiphile vaccine induces potent cellular and humoral immunity to SARS-CoV-2

**DOI:** 10.1126/sciadv.abe5819

**Published:** 2021-02-05

**Authors:** Martin P. Steinbuck, Lochana M. Seenappa, Aniela Jakubowski, Lisa K. McNeil, Christopher M. Haqq, Peter C. DeMuth

**Affiliations:** Elicio Therapeutics, One Kendall Square, Suite 14303, Cambridge, MA 02139, USA.

## Abstract

The profound consequences of severe acute respiratory syndrome coronavirus 2 (SARS-CoV-2) mandate urgent development of effective vaccines. Here, we evaluated an Amphiphile (AMP) vaccine adjuvant, AMP-CpG, composed of diacyl lipid–modified CpG, admixed with the SARS-CoV-2 Spike-2 receptor binding domain protein as a candidate vaccine (ELI-005) in mice. AMP modification efficiently delivers CpG to lymph nodes, where innate and adaptive immune responses are generated. Compared to alum, immunization with AMP-CpG induced >25-fold higher antigen-specific T cells that produced multiple T helper 1 (T_H_1) cytokines and trafficked into lung parenchyma. Antibody responses favored T_H_1 isotypes (IgG2c and IgG3) and potently neutralized Spike-2-ACE2 receptor binding, with titers 265-fold higher than natural convalescent patient COVID-19 responses; T cell and antibody responses were maintained despite 10-fold dose reduction in Spike antigen. Both cellular and humoral immune responses were preserved in aged mice. These advantages merit clinical translation to SARS-CoV-2 and other protein subunit vaccines.

## Teaser

Lymph node–targeting adjuvant AMP-CpG enhances SARS-CoV-2 RBD vaccine-induced cellular and humoral immune responses.

## INTRODUCTION

The pandemic of coronavirus disease 2019 (COVID-19), caused by severe acute respiratory syndrome coronavirus 2 (SARS-CoV-2), has resulted in worldwide social and economic consequences as well as substantial health care challenges. The severity of COVID-19 ranges from no symptoms to mild or moderate flu-like symptoms in approximately 80% of cases, to severe pneumonia and acute respiratory distress syndrome in up to 20% of patients (*1*). Conservative estimates of 1% case fatality predict more than 40 million global deaths (*2*). Unlike related betacoronaviruses, SARS and Middle East respiratory syndrome (MERS), the more efficient person-to-person transmission and prevalence of asymptomatic infection with SARS-CoV-2 has required public health mitigations including quarantine, contact tracing, face masks, and social distancing to reduce morbidity and mortality. Therefore, rapid development of effective vaccines is urgently needed.

An optimal SARS-CoV-2 vaccine should generate potent T cell immunity alongside neutralizing antibody responses. Recovery from SARS-CoV-2 infection without mechanical ventilation is significantly associated with elevated T cell levels (*3*–*5*), and T cell responses without humoral responses are sufficient for COVID-19 resolution (*6*). Conversely, COVID-19 death is associated with reduced T cells (*7*), with lymphocyte subset analyses implicating deficiency in both CD3^+^CD4^+^ and CD3^+^CD8^+^ cells (*3*). Like SARS-CoV-2, lethal MERS and SARS coronavirus infections were characterized by poor or absent T cell responses (*8*). However, individuals who recovered from SARS had detectable memory T cells 17 years later (*9*). Optimal SARS-CoV-2 vaccines must also produce a T helper 1 (T_H_1)–biased response since previous SARS (*10*) and MERS (*11*) vaccine candidates exacerbated lung disease associated with T_H_2 responses or as a result of antibody-dependent enhancement of viral entry. In addition, an optimal SARS-CoV-2 vaccine must be effective across age groups, especially in the elderly. COVID-19 mortality is frequent in patients >70 years of age, coinciding with an age-related decline in immune function and comorbidities including hypertension and diabetes (*12*).

**Fig. 1:**
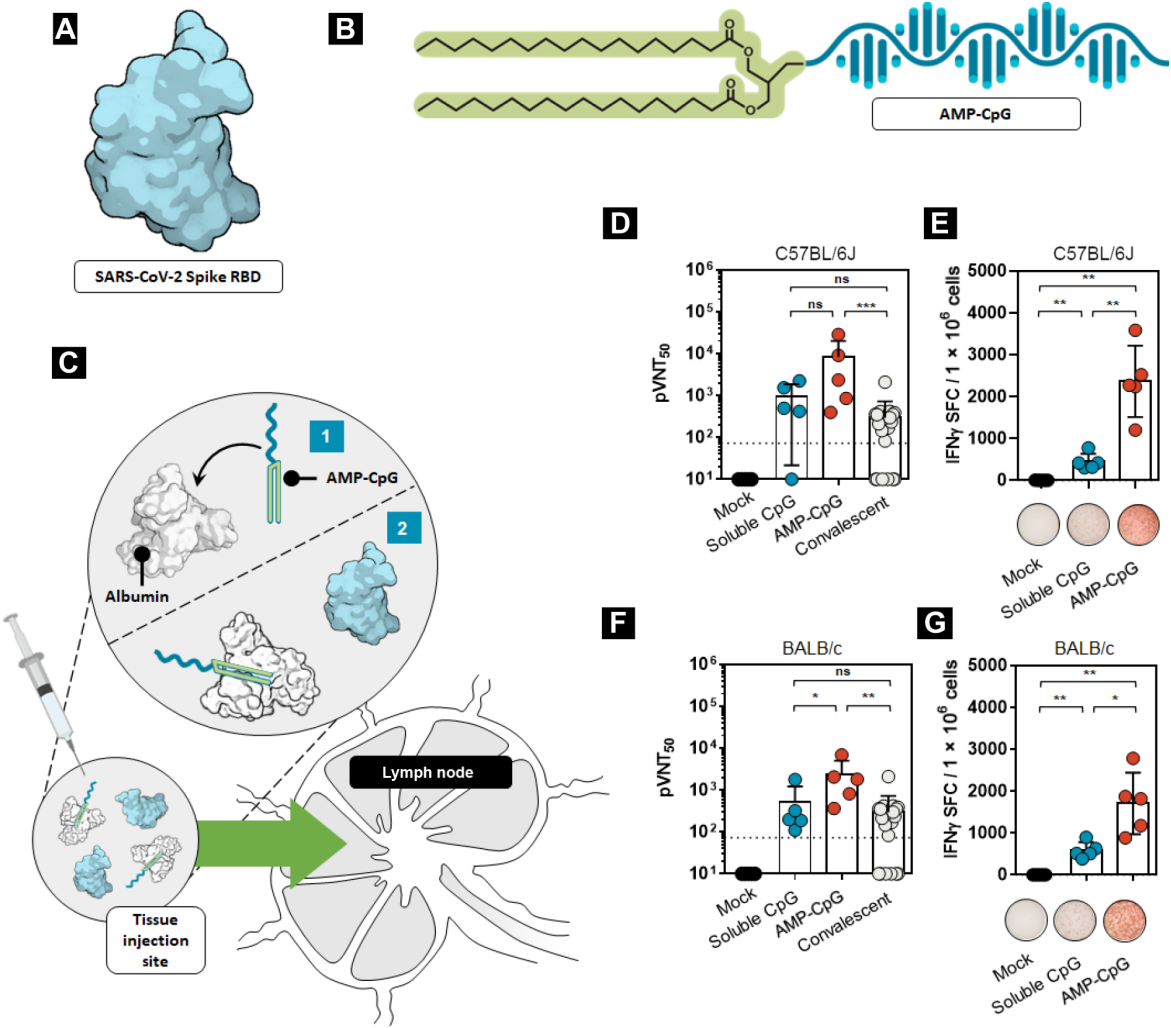
A lymph node–targeted subunit vaccine elicits enhanced humoral and cellular immunity to SARS-CoV-2. Protein subunit immunization was conducted with (**A**) recombinant Spike RBD and (**B**) AMP-CpG using a repeat-dose regimen. (**C**) Mechanism of AMP-CpG–targeted delivery to the lymph nodes. Following subcutaneous injection, (1) AMP-CpG binds to endogenous tissue-resident albumin and (2) albumin chaperones AMP-CpG into the lymph nodes. Co-injected Spike RBD comigrates into lymph nodes. (**D** to **G**) C57BL/6 or BALB/C mice (*n* = 5 per group) were immunized on days 0, 14, 28, and 42 with 10 μg of Spike RBD protein admixed with 1 nmol soluble CpG or AMP-CpG, and humoral and T cell responses were analyzed on day 49. Humoral and cellular immune responses were assessed in (D and E) C57BL/6 and (F and G) BALB/C mice (*n* = 5 per group) using (D and F) pseudovirus neutralization assays and (E and G) splenocyte IFNγ ELISpot assays, respectively. Pseudovirus neutralization activity was compared to sera/plasma from a cohort of 22 convalescent humans who had recovered from SARS-CoV-2 infection. Values depicted are means ± standard deviation. Non-detected values are shown on the baseline; *P < 0.05; **P < 0.01; ***P < 0.001; by two-sided Mann-Whitney test; ns, not significant. Pseudovirus LOD (indicated by the dotted line) was determined as mean + 90% confidence interval (CI) calculated for mock treatment.

We are evaluating an Amphiphile (AMP) adjuvant that efficiently accumulates in lymph nodes where protective immune responses are orchestrated. Following subcutaneous injection, components of subunit vaccines can be cleared through cell-mediated trafficking or passive uptake to the bloodstream or through lymphatic vessels; the passive clearance route is governed by molecular weight (*13*). Low–molecular weight compounds (<20 kDa) pass efficiently through the basement membrane and capillary endothelial tight junctions to enter the blood, which circulates 10-fold faster than lymph flow, leading to rapid systemic distribution to immunologically irrelevant or tolerizing sites, such as the liver (*13*, *14*). However, larger proteins such as albumin (65 kDa) almost exclusively transit from subcutaneous tissue into lymph (*14*). The AMP technology exploits this physiology using a molecular structure that conjugates adjuvants or antigens to an albumin binding moiety (such as a diacyl lipid; Fig. 1B), facilitating physical association with albumin to enable improved lymph node biodistribution (Fig. 1C). The resulting conjugate is referred to as AMP-CpG. Unlike AMP-CpG, soluble CpG traffics poorly to lymphatics, instead of entering the blood and rapidly distributing to irrelevant tissues, dampening the adjuvant effect (*15*). In contrast, AMP-CpG was previously shown to “hitchhike” on albumin to access the lymph and then draining lymph nodes, where it accumulates in key antigen-presenting cells, leading to a 30- to 50-fold increase in T cell and antibody responses to whole recombinant protein or peptide vaccines in mice with a concomitant reduction in systemic cytokine levels relative to soluble comparators (*15*, *16*). These studies reported no evidence of immune responses specific to endogenous albumin.

The AMP technology is currently being developed for therapeutic cancer vaccines to enhance lymphatic delivery of antigenic peptides, adjuvants, CAR-T activators for hematological and solid tumors (*17*), cytokines, and immunomodulatory agents. On the basis of preclinical work to support the oncology indications and the work presented in this communication, we hypothesize that AMP-CpG will combine with SARS-CoV-2 antigens to provide a safe and effective SARS-CoV-2 vaccine.

Here, we describe the immunogenicity of AMP-CpG paired with SARS-CoV-2 Spike receptor binding domain protein (hereafter referred to as Spike RBD), which is of sufficient size (approximately 34 kDa) to traffic to lymph nodes after subcutaneous injection (*13*). Spike RBD is the target of neutralizing antibodies that block the interaction between SARS-CoV-2 and its angiotensin converting enzyme 2 (ACE2) human receptor (*18*). Several groups have shown that Spike-based antigens serve as targets for neutralizing humoral responses to SARS (*10*), MERS (*11*), and SARS-CoV-2 in mice, rats, primates, and humans (*19*, *20*). Likewise, Spike antigens contain T cell epitopes, generating cell-mediated immunity in SARS (*21*), MERS (*8*), and SARS-CoV-2 (*5*, *22*, *23*).

We evaluated T cell immunity in splenocytes, peripheral blood, cells from perfused lung tissues, and bronchoalveolar lavage (BAL) fluid. We also measured humoral responses specific to Spike RBD through enzyme-linked immunosorbent assays (ELISAs), ACE2 competition ELISA, and pseudovirus neutralization assays. Furthermore, we compared immune responses following administration of a range of antigen concentrations, and we evaluated immune responses in aged mice. The data show that immunization against Spike RBD with AMP-CpG elicits T_H_1-biased polyfunctional cellular and humoral responses in young and aged mice. The AMP-CpG vaccine (ELI-005) could offer an optimal vaccine for general clinical use.

## RESULTS

### Formulation design

For assessment of the AMP technology as a possible vaccine for SARS-CoV-2, we evaluated AMP-CpG (consisting of a diacyl lipid conjugated to CpG DNA) admixed with Spike RBD (Fig. 1, A and B) in a repeat-dose immunization regimen. Following subcutaneous injection, AMP-CpG binds to endogenous tissue-resident albumin, which then travels to lymph nodes where it effectively colocalizes with lymph node–resident immune cells (Fig. 1C) (*15*). Initial assessments in C57BL/6J and BALB/C mice receiving immunization containing AMP-CpG produced an 8- to 28-fold higher pseudovirus neutralizing titer than natural antibody responses in human convalescent serum (obtained from recovered patients with COVID-19; Fig. 1, D and F), indicating the potential for AMP-CpG to produce neutralizing antibody responses more potent than natural immunity. By comparison, animals immunized with a dose-matched regimen containing unmodified (soluble) CpG produced neutralizing titers comparable to those observed in human convalescing patients. Splenocyte ELISpot assays showed that compared with soluble CpG, mice immunized with Spike RBD admixed with AMP-CpG elicited approximately fourfold greater frequencies of antigen-specific functional T cells, producing interferon-γ (IFNγ) upon stimulation with Spike-derived overlapping peptides (Fig. 1, E and G).

### Cellular immune response

#### Splenocytes and peripheral blood

**Fig. 2:**
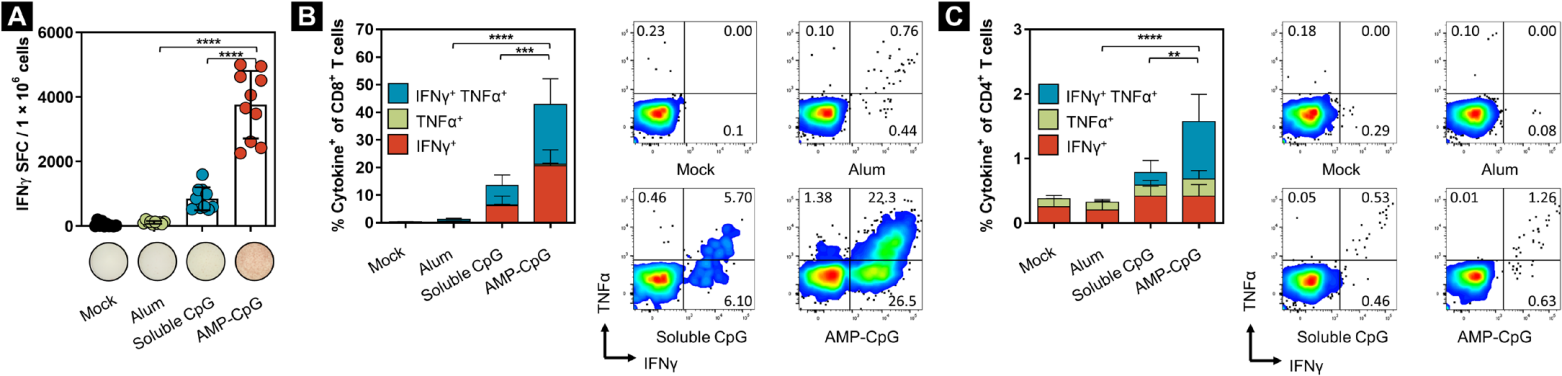
Vaccination with AMP-CpG elicits potent Spike RBD–specific CD8 and CD4 T cells in spleen and blood. C57BL/6 mice (*n* = 10 per group) were immunized on days 0, 14, and 28 with 10 μg of Spike RBD protein admixed with 100 μg of alum or 1 nmol soluble CpG or AMP-CpG, and T cell responses were analyzed on day 35. (**A**) Splenocytes were restimulated with overlapping Spike RBD peptides and assayed for IFNγ production by ELISpot assay. Shown are representative images of ELISpot and frequency of IFNγ SFCs per 1 × 10^6^ splenocytes. (**B** and **C**) Peripheral blood cells were restimulated with overlapping Spike RBD peptides and assayed by flow cytometry for intracellular cytokine production to detect antigen-specific T cell responses. Shown are frequencies of IFNγ, TNFα, and double-positive T cells among (B) CD8^+^ and (C) CD4^+^ T cells, with corresponding representative flow cytometry plots. *n* = 10 mice per group. Values depicted are means ± standard deviation. **P < 0.01; ***P < 0.001; ****P < 0.0001 by two-sided Mann-Whitney test applied to cytokine^+^ T cell frequencies.

To further characterize the contribution of a lymph node–targeted adjuvant on Spike RBD–specific immunity, we compared AMP-CpG to dose-matched formulations of antigen admixed with either soluble CpG (representative of an adjuvant with poor lymph node uptake) or alum (as a benchmark clinical adjuvant). We assessed cytokine-producing cells in splenocytes and peripheral blood from C57BL/6J mice on day 35. Mice immunized with AMP-CpG had substantially higher IFNγ spot-forming cells (SFCs) than mice dosed with soluble CpG, alum, or mock (Fig. 2A). Approximately 43% of CD8^+^ T cells derived from peripheral blood in AMP-CpG–immunized mice were cytokine^+^ in response to stimulation with overlapping RBD peptides [IFNγ, tumor necrosis factor–α (TNFα), or double-positive T cells]; in comparison, approximately 13% and <2% of CD8^+^ T cells were cytokine-producing for soluble CpG-immunized mice and alum-immunized mice, respectively (Fig. 2B). A similar trend was observed for CD4^+^ T cells, though percentages were relatively smaller: approximately 1.5% of T cells in peripheral blood from AMP-CpG–immunized mice were cytokine-producing compared with <1% in CpG-immunized mice and <0.5% for alum-immunized mice and mock-immunized mice (Fig. 2C). Evaluation of cytokine-positive T cell responses in mice immunized with only two doses of AMP-CpG vaccine indicated that comparably high frequencies of RBD-specific CD8^+^ and CD4^+^ T cell responses were induced in the blood (fig. S1).

#### Perfused lung tissue

**Fig. 3:**
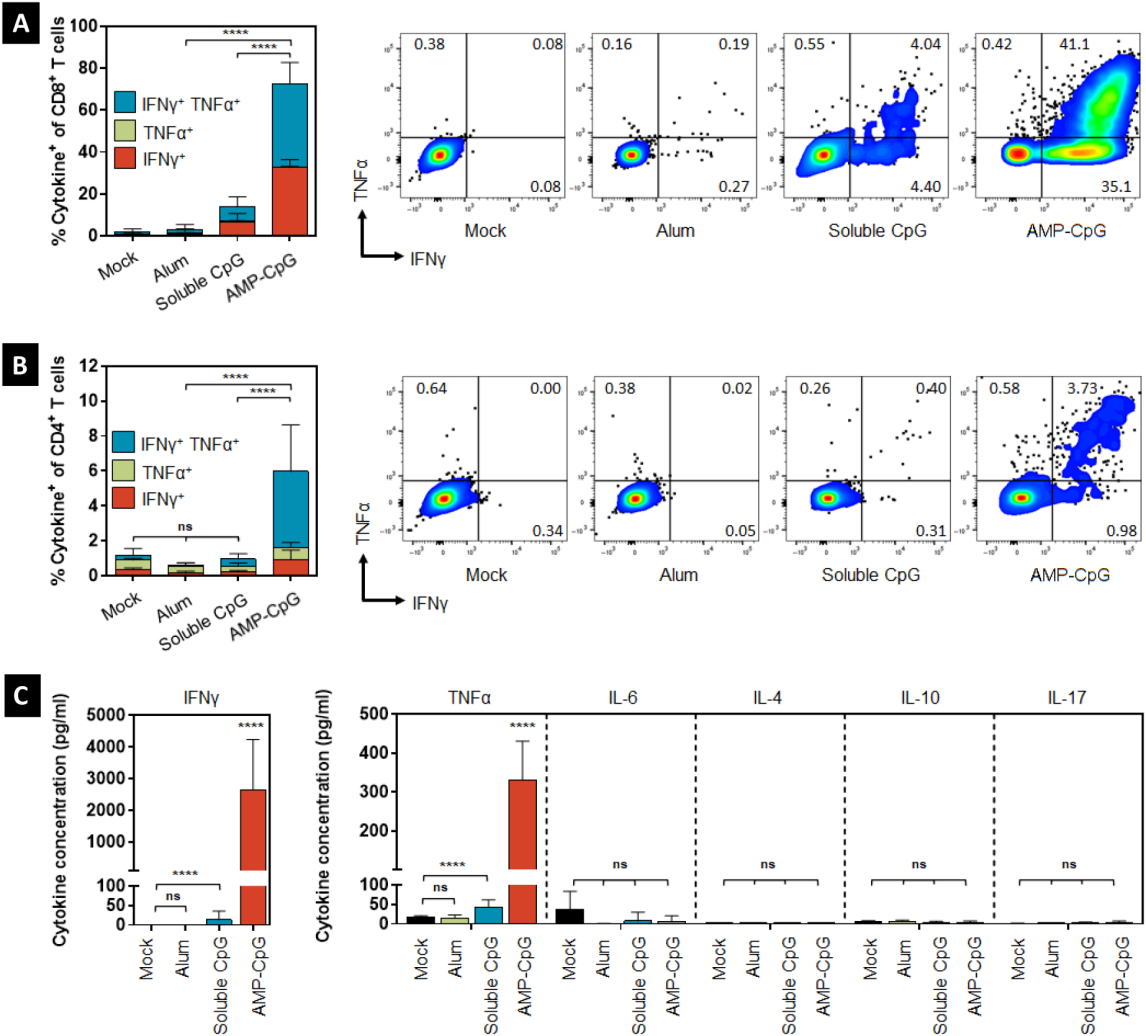
Vaccination with AMP-CpG elicits potent lung Spike RBD–specific CD8 and CD4 T cells. C57BL/6 mice (*n* = 10 per group) were immunized on days 0, 14, and 28 with 10 μg of Spike RBD protein admixed with 100 μg of alum or 1 nmol soluble CpG or AMP-CpG, and T cell responses were analyzed on day 35. (**A**) Cells collected from perfused lung tissue were restimulated with overlapping Spike RBD peptides and assayed for cytokine production to detect antigen-specific T cell responses. Shown are frequencies (means ± standard deviation) of IFNγ, TNFα, and double-positive T cells among (A) CD8^+^ and (**B**) CD4^+^ T cells, with corresponding representative flow cytometry plots. (**C**) Cytokine concentration in supernatants from restimulated lung cells. *n* = 10 mice per group. Values depicted are means ± standard deviation. ****P < 0.0001 by two-sided Mann-Whitney test applied to cytokine^+^ T cell frequencies or cytokine concentrations.

To determine whether immunization could induce pulmonary T cell responses at a site of likely SARS-CoV-2 exposure, we evaluated the proportion of cytokine-producing cells from perfused lung tissue from C57BL/6J mice on day 35. As observed with responses assayed in peripheral blood, the proportion of cytokine-producing cells among CD8^+^ or CD4^+^ T cells was highest in AMP-CpG–immunized mice compared with soluble CpG, alum, or mock treatment (Fig. 3, A and B). T cells in the lung tissue had a greater proportion of the cytokine-producing cells than observed in peripheral blood. Observations in AMP-CpG–immunized mice showed that approximately 73% of CD8^+^ T cells from perfused lung tissues were cytokine-producing, with approximately 40% exhibiting polyfunctional secretion of both T_H_1 cytokines IFNγ and TNFα. By comparison, immunization with soluble CpG or alum induced >5- and >25-fold lower responses, respectively. Similar assessment of CD4^+^ T cells showed that only AMP-CpG–immunized animals generated responses above background, with approximately 6% of CD4^+^ T cells producing IFNγ and/or TNFα, again exhibiting strong polyfunctional effector functionality, with most of these cells able to produce both IFNγ and TNFα upon antigen stimulation. Comparable RBD-specific CD8^+^ and CD4^+^ T cell responses resulted from a two-dose regimen (fig. S1). These results show that the more potent lymph node action of AMP-CpG induces enhanced frequency of antigen-specific T cells in the lung, a primary site of initial viral exposure.

To more comprehensively understand the T_H_1/T_H_2/T_H_17 profile of the elicited T cell responses, we used a multiplexed cytokine assay to assess various cytokine concentrations from supernatants of cells collected from perfused lungs following stimulation with Spike-derived overlapping peptides. AMP-CpG–immunized mice exhibited a T_H_1 effector profile consistent with prior assessment by flow cytometry with IFNγ and TNFα concentrations that were significantly higher than cohorts immunized with the other adjuvants (soluble CpG and alum) or mock: The IFNγ concentration was at least 200-fold higher than observed with the other adjuvants or mock, and the TNFα concentration was at least 7-fold higher (Fig. 3C). Concentrations of common T_H_2- or T_H_17-associated cytokines interleukin-4 (IL-4), IL-6, IL-10, and IL-17 were undetectable for all cohorts. These results further demonstrate the greatly enhanced potency and T_H_1 bias in T cells elicited through immunization with AMP-CpG compared with either soluble CpG or alum.

#### BAL fluid

Together with T cells in the lung parenchyma, BAL T cells may be ideally positioned to rapidly respond to prevent or control nascent viral infection. To further evaluate whether lung T cell responses induced by immunization could localize into lung secretions, we collected BAL fluid from C57BL/6J immunized mice on day 35 and determined T cell numbers and phenotypic characteristics. We found significantly more CD8^+^ T cells in BAL fluid of AMP-CpG immunized mice than other treatment groups (fig. S2A). In addition, a significantly lower proportion of cells detected in the BAL collected from AMP-CpG–immunized animals exhibited a naïve phenotype (CD44^−^, CD62L^+^; fig. S2B) with a corresponding increase in the frequency of effector memory phenotype (T_EM_; CD44^+^, CD62L^−^; fig. S2C). The CD4^+^ T cell count was enhanced relative to mock treatment and generally similar across all treatment groups (fig. S2D), but the AMP-CpG cohort showed evidence that a significantly greater proportion of the BAL CD4^+^ T cells had differentiated from naïve to T_EM_ phenotype than in the other treatment groups (fig. S2F). The improved numbers and phenotype of BAL T cells present in AMP-CpG–immunized animals demonstrate a greater potential for early immunological detection and control at the point of viral exposure.

### Humoral responses

#### Neutralizing antibody

**Fig. 4:**
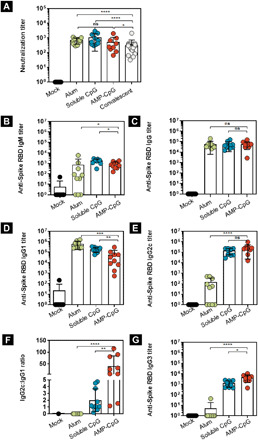
Vaccination with AMP-CpG elicits potent T_H_1-biased Spike RBD humoral responses. C57BL/6 mice (*n* = 10 per group) were immunized on days 0, 14, and 28 with 10 μg of Spike RBD protein admixed with 100 μg of alum or 1 nmol soluble CpG or AMP-CpG, and humoral responses were analyzed on day 7 or day 35. Humoral responses specific to Spike RBD were assessed in serum from immunized animals by pseudovirus neutralization or ELISA assay. Shown are (**A**) surrogate neutralization endpoint titers on day 35, (**B** to **G**) endpoint titers, and endpoint titer ratios determined by ELISA for (B) immunoglobulin M (IgM) on day 7, (C) IgG, (D) IgG1, and (E) IgG2c on day 35, (F) IgG2c:IgG1 ratio, and (G) IgG3 on day 35. Pseudovirus neutralization activity was compared to sera/plasma from a cohort of 22 convalescent humans who had recovered from SARS-CoV-2 infection. *n* = 10 mice per group. Values depicted are means ± standard deviation. Non-detected values are shown on the baseline; *P < 0.05; **P < 0.01; ***P < 0.001; ****P < 0.0001 by two-sided Mann-Whitney test.

Neutralizing antibody responses to Spike RBD are generated in convalescing patients and are a primary goal for vaccines that would prevent infection or limit its severity. We assessed antibody activity through measurement of inhibition of the Spike-RBD-ACE2 interaction in an ELISA-based ACE2 competition assay. Results for serum collected on day 35 for cohorts of immunized C57BL/6J mice are shown in Fig. 4A. Comparable levels of RBD ACE2 receptor blocking activity were induced in animals immunized with AMP-CpG, soluble CpG, and alum. Comparison with samples obtained from a cohort of convalescent humans showed that the vaccine-induced responses were significantly higher than those generated through response to natural infection.

#### IgM/IgG

Seven days after the initial immunization, all cohorts—except the control receiving mock immunization—showed robust Spike RBD-specific immunoglobulin M (IgM) responses (Fig. 4B), which underwent isotype switching to produce immunoglobulin G (IgG) responses with similar titer following subsequent boosting immunization (Fig. 4C). Assessment of IgA in BAL fluid showed no detectable RBD-specific responses in any treatment group. No anti-albumin IgG responses were detected in the serum of any treatment group.

#### IgG subclasses

To assess T_H_1/T_H_2 bias in the Spike RBD-specific IgG response elicited through immunization, we evaluated the IgG subclasses present and found that mice immunized with AMP-CpG or soluble CpG had significantly lower T_H_2-associated IgG1 titers (approximately 3- to 10-fold) than mice immunized with alum (Fig. 4D). The reverse was true for T_H_1-associated IgG2c; titers were significantly higher (approximately 10- to 1000-fold) for mice immunized with AMP-CpG or soluble CpG (Fig. 4E). The ratio of IgG2c:IgG1 titer indicated a strong bias toward T_H_1 for AMP-CpG–immunized animals, while soluble CpG and alum produced a balanced T_H_1/T_H_2 profile or T_H_2-dominant response, respectively (Fig. 4F). Further analysis showed that AMP-CpG–immunized animals produced significantly higher IgG3 titers than either soluble CpG (approximately 3-fold) or alum (>800-fold) treatment groups consistent with the observed T_H_1 bias resulting from AMP-CpG immunization (Fig. 4G).

### Antigen dose sparing

Given the need for immunization against SARS-CoV-2 on a global scale, and the observed potency of AMP-CpG for induction of potent neutralizing antibodies and CD8^+^ and CD4^+^ T cells in peripheral blood, lung, and BAL, we sought to determine whether dose sparing could produce similar immune responses with reduced doses of Spike RBD antigen. Here, we studied the immune response generated by repeat-dose immunization with AMP-CpG admixed with three different dose levels of Spike RBD (1, 5, and 10 μg) and compared these to responses induced by immunization with soluble CpG and alum admixed with 10 μg of antigen.

**Fig. 5:**
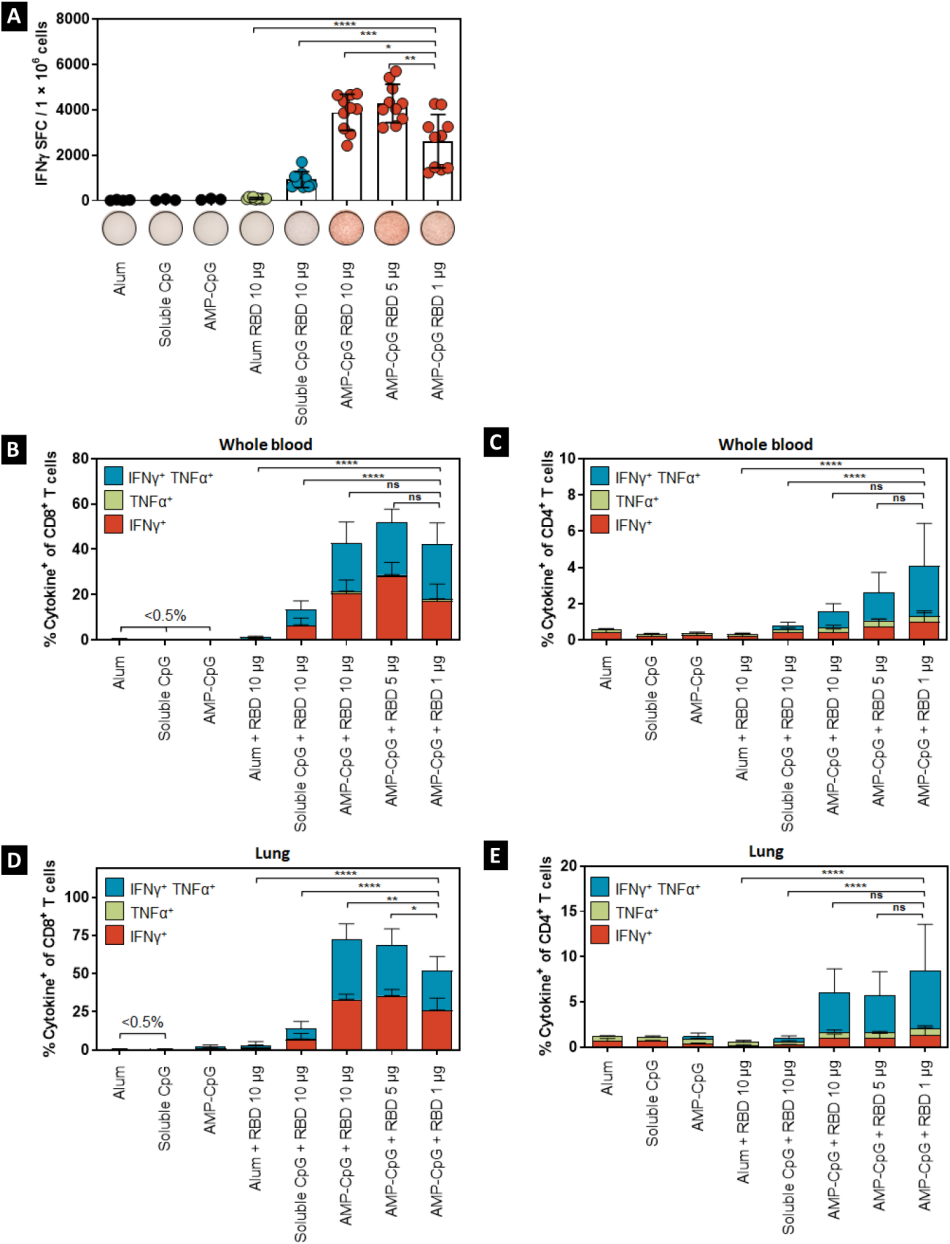
Vaccination with AMP-CpG enables dose sparing of Spike RBD to elicit cellular immunity. C57BL/6 mice (*n* = 10 per group) were immunized on days 0, 14, and 28 with 10 μg of Spike RBD protein admixed with 100 μg of alum or 1 nmol soluble CpG. Comparator animals were dosed with 1, 5, or 10 μg of Spike RBD admixed with 1 nmol AMP-CpG. Control animals were dosed with adjuvant alone. T cell responses were analyzed on day 35. (**A**) Splenocytes were restimulated with overlapping Spike RBD peptides and assayed for IFNγ production by ELISpot assay. Shown are representative images of ELISpot and frequency of IFNγ SFCs per 1 × 10^6^ splenocytes. Cells collected from (**B** and **C**) peripheral blood and (**D** and **E**) perfused lung tissue were restimulated with overlapping Spike RBD peptides and assayed for intracellular cytokine production to detect antigen-specific T cell responses. Shown are frequencies of IFNγ, TNFα, and double-positive T cells among (B) peripheral blood CD8^+^ and (C) CD4^+^ T cells, and (D) lung CD8^+^ and (E) CD4^+^ T cells. *n* = 10 mice per group. Values depicted are means ± standard deviation. *P < 0.05; **P < 0.01; ***P < 0.001; ****P < 0.0001 by two-sided Mann-Whitney test applied to cytokine^+^ T cell frequencies.

We evaluated T cell responses on day 35 in spleen, peripheral blood, and lung tissues. The results showed that the frequency of IFNγ-producing cells in splenocytes collected from AMP-CpG–immunized C57BL/6J mice tended to increase with antigen concentration, but, even at the lowest antigen dose admixed with AMP-CpG, the frequency of IFNγ-producing cells was significantly higher than observed in cohorts that received the highest antigen dose (10 μg) with either soluble CpG (approximately 6-fold) or alum (>40-fold) (Fig. 5A). In both peripheral blood (Fig. 5, B and C, and fig. S3) and lung tissue (Fig. 5, D and E, and fig. S4), the percentage of CD8^+^ and CD4^+^ T cells producing cytokine was significantly higher for AMP-CpG–treated mice at any concentration of antigen compared with the other adjuvants tested. Notably, no significant decrease in the frequency of cytokine-producing CD8^+^ or CD4^+^ T cells was observed in the peripheral blood of animals immunized with AMP-CpG admixed with antigen at 10-, 5-, or 1-μg dose levels as these were maintained at approximately 40 to 50% of CD8^+^ and 2 to 4% of CD4^+^ T cells. While a decreasing trend was observed in the frequency of lung cytokine-producing CD8^+^ T cells in AMP-CpG–immunized animals, even the 1-μg dose level produced frequencies >3- or >30-fold higher than animals immunized with soluble CpG or alum, respectively. This indicates a significant potential for AMP-CpG to enable at least 10-fold dose sparing of RBD protein.

**Fig. 6:**
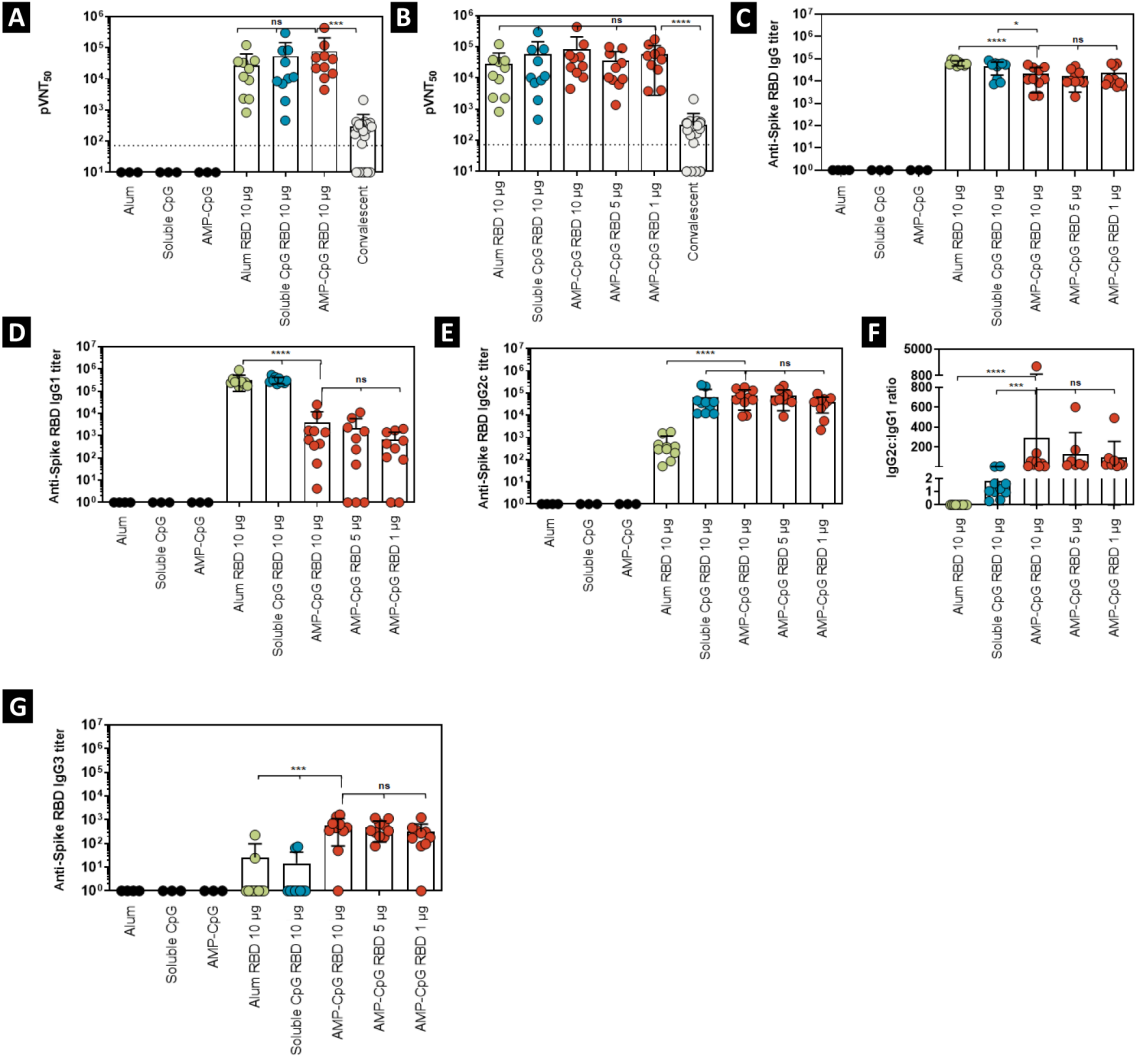
Vaccination with AMP-CpG enables dose sparing of Spike RBD to elicit humoral immunity. C57BL/6 mice (*n* = 10 per group) were immunized on days 0, 14, and 28 with 10 μg of Spike RBD protein admixed with 100 μg of alum or 1 nmol soluble CpG. Comparator animals were dosed with 1, 5, or 10 μg of Spike RBD admixed with 1 nmol AMP-CpG. Control animals were dosed with adjuvant alone. Humoral responses were analyzed on day 35. Humoral responses specific to Spike RBD were assessed in serum from immunized animals by ELISA or pseudovirus neutralization assay. Shown are (**A** and **B**) pseudovirus neutralization ID_50_ (pVNT_50_) on day 35, (**C** to **G**) endpoint titers, and endpoint titer ratios determined for (C) IgG, (D) IgG1, (E) IgG2c, (F) IgG2c:IgG1 ratio, and (G) IgG3 on day 35. Pseudovirus neutralization activity was compared to a cohort of 22 convalescent humans who had recovered from SARS-CoV-2 infection. *n* = 10 mice per group. Values depicted are means ± standard deviation. Non-detected values are shown on the baseline; *P < 0.05; ***P < 0.001; ****P < 0.0001 by two-sided Mann-Whitney test. Pseudovirus LOD (indicated by the dotted line) was determined as mean + 90% CI calculated for mock treatment.

On day 35 following repeat-dose immunization, we assessed the induction of Spike RBD–specific antibody responses among the AMP-CpG–immunized animals at each specified Spike RBD dose level for comparison to responses generated by immunization with either soluble CpG or alum at the 10-μg dose. Neutralizing activity was assessed through measurement of pseudovirus neutralization titers at the half-maximal inhibitory dilution (pVNT_50_). Similar levels of pseudovirus neutralization titers were observed for all treatment groups, at levels that were 265-, 184-, or 94-fold greater than those observed in convalescent human samples, for AMP-CpG–, soluble CpG–, and alum-immunized mice, respectively (Fig. 6A). Notably, these levels were maintained in animals immunized with AMP-CpG at lower Spike RBD dose levels with a mean pVNT_50_ of at least 115-fold greater than those measured in recovering patients with COVID-19 (Fig. 6B).

Total IgG titers were similar among the groups administered AMP-CpG, and these were reduced approximately twofold in comparison to titers measured among groups dosed with either soluble CpG– or alum-adjuvanted vaccines (Fig. 6C). Isotype analysis demonstrated similar trends to those initially observed in comparison at the 10-μg dose level (Fig. 4, C to G). Alum and soluble CpG immunization produced significantly higher T_H_2-associated IgG1 titers (approximately 100-fold) compared to all Spike RBD dose levels admixed with AMP-CpG (Fig. 6D). T_H_1-associated IgG2c levels were elevated approximately 20-fold in all AMP-CpG–immunized animals compared with soluble CpG– and alum-immunized groups, with no significant difference observed with reduced Spike RBD dose level (Fig. 6E). These trends were further evident in the comparison of IgG2c:IgG1 titer ratio (Fig. 6F), where AMP-CpG–containing regimens induced highly T_H_1-dominant isotype profile (IgG2c:IgG1 > 90), compared with more balanced and T_H_2-skewed responses in soluble CpG (IgG2c:IgG1 approximately 2)– and alum (IgG2c:IgG1 <1)–vaccinated animals, respectively. Last, only animals immunized with AMP-CpG showed evidence of Spike RBD–specific IgG3 titers, with comparable levels detected among all Spike RBD dose levels (approximately 500-fold over background). Furthermore, AMP-CpG–immunized animals produced significantly higher IgG3 titers than either soluble CpG (approximately 40-fold) or alum (>20-fold) treatment groups consistent with the observed T_H_1 bias resulting from AMP-CpG immunization (Fig. 6G). Together, these data demonstrate the potential for AMP-CpG to enable at least 10-fold dose sparing of Spike RBD antigen for induction of neutralizing, high-titer, and T_H_1-biased antibody responses against Spike RBD. While soluble CpG and alum induced marginally higher total IgG responses, these did not result in significant differences in neutralizing activity compared to AMP-CpG immunization.

### Immune response in aged mice

Protective immunization in at-risk populations is of utmost importance. Specifically, COVID-19 has demonstrated a higher incidence of severe disease and mortality among the elderly coincident with a general decline in immune function associated with aging (*24*, *25*). To assess the potential of AMP-CpG to elicit effective immunity in the setting of deficient baseline immune function, we immunized aged mice to compare immune responses generated from vaccines containing AMP-CpG, soluble CpG, and alum. We further investigated the effect of Spike RBD protein dose in this model to determine the potential for AMP-CpG to enable antigen dose sparing similar to that observed in studies of young healthy mice.

**Fig. 7:**
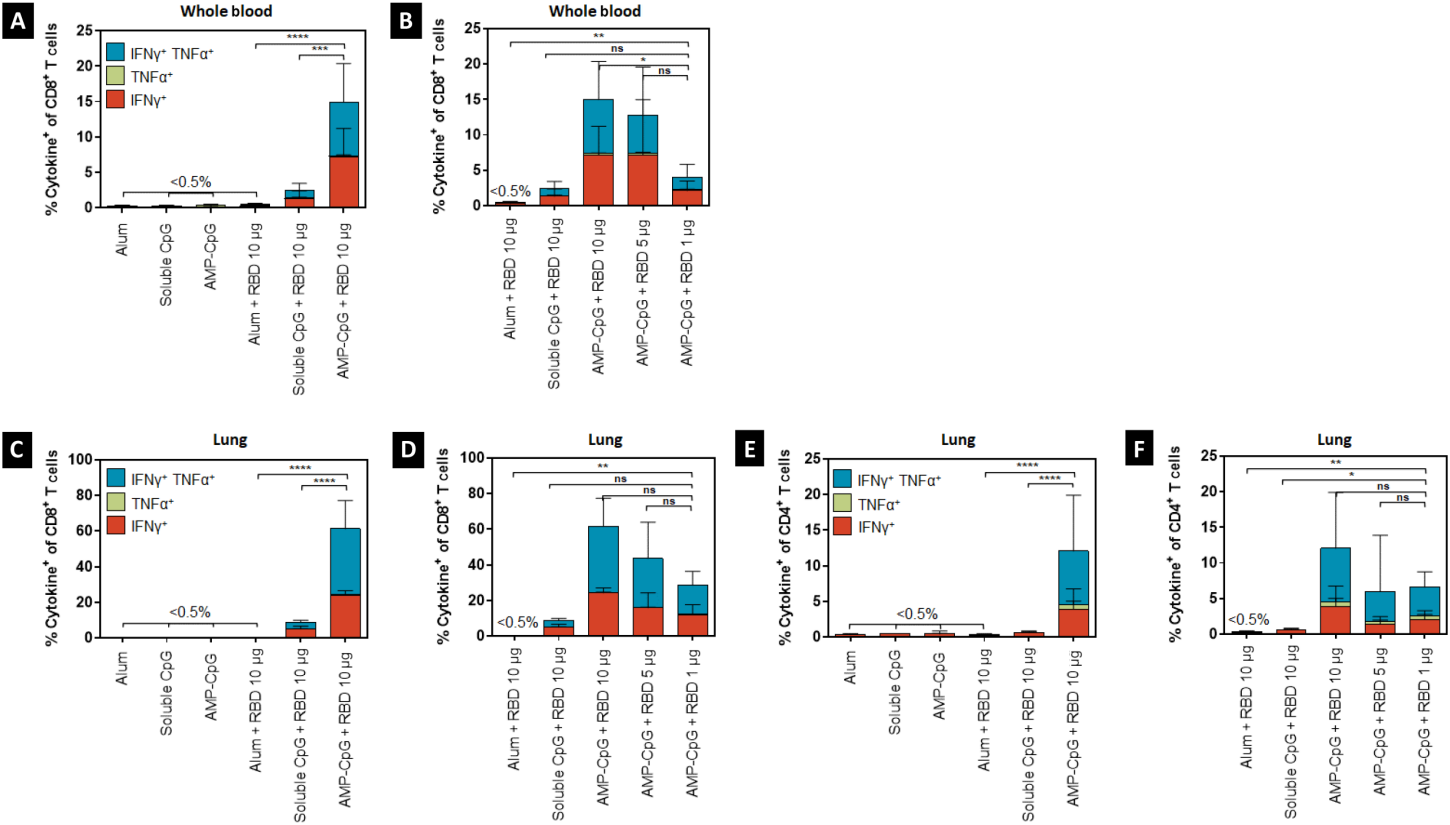
Vaccination with AMP-CpG in aged mice enables dose sparing of Spike RBD to elicit cellular immunity. Thirty-seven-week-old C57BL/6 mice (*n* = 10 per group) were immunized on days 0, 14, and 28 with 10 μg of Spike RBD protein admixed with 100 μg of alum or 1 nmol soluble CpG. Comparator animals were dosed with 1, 5, or 10 μg of Spike RBD admixed with 1 nmol AMP-CpG. Control animals were dosed with adjuvant alone. T cell responses were analyzed on days 21 and 35. Cells collected from (**A** and **B**) peripheral blood on day 21 and (**C** to **F**) perfused lung tissue on day 35 were restimulated with overlapping Spike RBD peptides and assayed for intracellular cytokine production to detect antigen-specific T cell responses. Shown are frequencies of IFNγ, TNFα, and double-positive T cells among (A and B) peripheral blood CD8^+^, and (C and D) lung CD8^+^ and (E and F) CD4^+^ T cells. *n* = 10 mice per group. Values depicted are means ± standard deviation. *P < 0.05; **P < 0.01; ***P < 0.001; ****P < 0.0001 by two-sided Mann-Whitney test applied to cytokine^+^ T cell frequencies.

We evaluated T cell responses in aged mice after repeat-dose immunization with comparator vaccines on days 21 and 35. Assessment on day 21 of cytokine-producing CD8^+^ T cells in peripheral blood following Spike-derived overlapping peptide stimulation showed that AMP-CpG induced potent responses (approximately 15% of CD8^+^ T cells), greatly outperforming soluble CpG (approximately 2.5% of CD8^+^ T cells) and alum (<0.5% of CD8^+^ T cells) (Fig. 7A). Although these responses were reduced approximately 2-fold compared to those observed in young healthy mice, they nonetheless exceeded those generated by the soluble CpG and alum comparators by 6- and 30-fold, respectively. AMP-CpG immunization further enabled comparable responses at 10- and 5-μg Spike RBD doses, and although responses at 1 μg were decreased, these still exceeded the response observed for alum (8-fold) and were similar to those generated through immunization with soluble CpG at the 10-μg dose level (Fig. 7B). These responses were durable as robust AMP-CpG vaccine–induced T cell responses were maintained at higher levels than alum or soluble CpG comparators in blood, spleen, and lung 42 days following the final immunization (fig. S5).

Analysis on day 35 of CD8^+^ and CD4^+^ T cells in lung tissue of aged mice showed a similar trend, with AMP-CpG–immunized animals producing high frequencies of cytokine-producing CD8^+^ and CD4^+^ T cells. Specifically, AMP-CpG immunization elicited highly polyfunctional T_H_1 cytokine production in approximately 60% of lung CD8^+^ T cells, approximately 7-fold and >230-fold higher than soluble CpG and alum immunization, respectively (Fig. 7C). Unlike the responses in peripheral blood, lung cytokine-producing CD8^+^ T cell frequencies did not decline in aged mice following AMP-CpG immunization relative to responses in young healthy animals and were maintained at statistically comparable levels in the 5- and 1-μg Spike RBD–dosed groups (Fig. 7D). Lung CD4^+^ T cells exhibited a similar hierarchy of response among the comparators (Fig. 7E) comparable to those observed in young healthy mice showing that AMP-CpG immunization can raise equally potent lung T cell responses in young and aged mice. As observed in lung T cell responses detected in young animals, AMP-CpG immunizations at the lowest concentration of Spike RBD (1 μg) outperformed both soluble CpG and alum at a 10-fold higher antigen dose (10 μg; Fig. 7F).

**Fig. 8:**
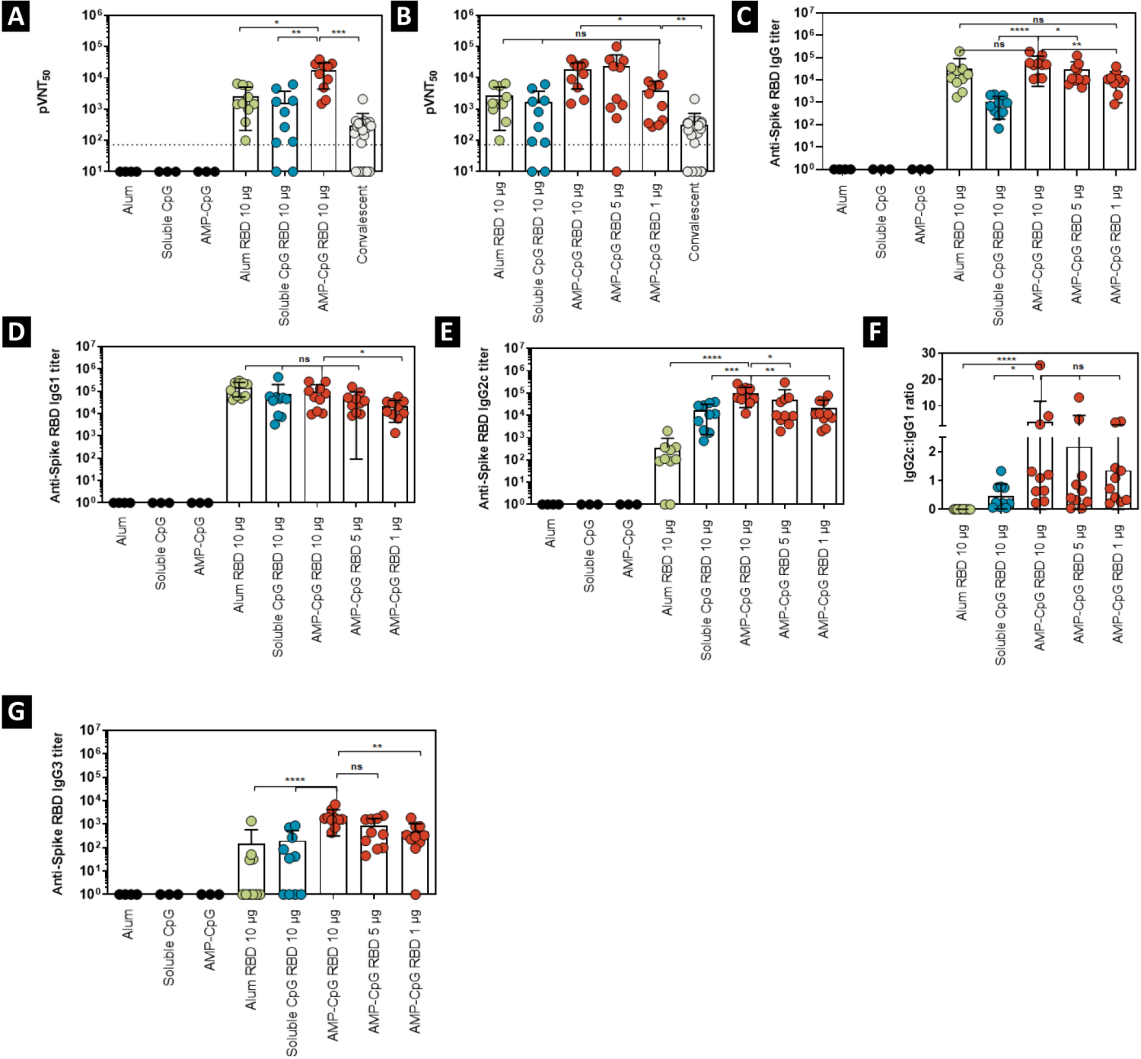
Vaccination with AMP-CpG in aged mice enables dose sparing of Spike RBD to elicit humoral immunity. Thirty-seven-week-old C57BL/6 mice (*n* = 10 per group) were immunized on days 0, 14, and 28 with 10 μg of Spike RBD protein admixed with 100 μg of alum or 1 nmol soluble CpG. Comparator animals were dosed with 1, 5, or 10 μg of Spike RBD admixed with 1 nmol AMP-CpG. Control animals were dosed with adjuvant alone. Humoral responses were analyzed on day 35. Humoral responses specific to Spike RBD were assessed in serum from immunized animals by ELISA or pseudovirus neutralization assay. Shown are (**A** and **B**) pseudovirus neutralization ID_50_ (pVNT_50_) on day 35, (**C** to **G**) endpoint titers and endpoint titer ratios determined for (C) IgG, (D) IgG1, (E) IgG2c, (F) IgG2c:IgG1 ratio, and (G) IgG3 on day 35. Pseudovirus neutralization activity was compared to serum/plasma from a cohort of 22 convalescent humans who had recovered from SARS-CoV-2 infection. *n* = 10 mice per group. Values depicted are means ± standard deviation. Non-detected values are shown on the baseline; *P < 0.05; **P < 0.01; ***P < 0.001; ****P < 0.0001 by two-sided Mann-Whitney test. Pseudovirus LOD (indicated by the dotted line) was determined as mean + 90% CI calculated for mock treatment.

Spike RBD–specific antibody responses were evaluated on day 35 after repeat-dose immunization with comparator vaccines in aged mice. Pseudovirus neutralization showed that AMP-CpG immunization at the 10-μg antigen dose level elicited enhanced neutralizing titers, at least 5-fold greater than those observed for dose-matched soluble CpG and alum comparators, and >50-fold greater than observed in human convalescent sera/plasma (Fig. 8A). Reduced doses of Spike RBD with AMP-CpG gave lower neutralizing titers that were comparable to soluble CpG and alum (Fig. 8B). Of particular interest was the equivalency of titers from animals immunized with 10 μg of Spike RBD with soluble CpG or alum relative to those receiving the lower 1-μg Spike RBD dose with AMP-CpG. Assessment of total IgG showed that AMP-CpG and alum produced comparable Spike RBD–specific IgG titers, both in excess of that generated in soluble CpG–immunized animals (Fig. 8C). Although a significant decline was observed in IgG titer with decreasing Spike RBD dose in AMP-CpG–immunized animals, there was no statistical difference between AMP-CpG with 1 μg of Spike RBD and alum with 10 μg of Spike RBD. Anti-Spike RBD serum IgG responses were stable up to day 70, 42 days after the final immunization (fig. S5). Isotype analysis yielded similar observations to those made in young healthy mice, with AMP-CpG driving more T_H_1, IgG2c-dominant responses compared with soluble CpG or alum, which yielded more balanced or T_H_1, IgG1-biased profiles (Fig. 8, D to G). No significant difference was observed among AMP-CpG–immunized animals at the varying dose levels of Spike RBD (Fig. 8F), although the strength of T_H_1 bias observed for AMP-CpG–immunized mice was reduced in aged mice relative to young healthy mice. As previously observed in young healthy mice, IgG3 titers were enhanced in AMP-CpG–immunized animals compared with soluble CpG or alum (Fig. 8G). Together, these results show the potential for AMP-CpG to elicit potent and functional Spike RBD–specific humoral immunity in aged mice beyond what was observed for soluble CpG or alum vaccine comparators while producing an optimal T_H_1-biased isotype profile and enabling at least 10-fold dose sparing of antigen.

## DISCUSSION

Development of a safe and effective SARS-CoV-2 vaccine is urgently needed considering the profound public health, economic, and social impacts of the COVID-19 global pandemic. Multiple parallel efforts to generate a COVID-19 vaccine are ongoing to improve the chance of success (*26*, *27*).

Emerging data from patients with SARS-CoV-2 along with earlier data from individuals with MERS and SARS infections have provided information about the natural immune response to COVID-19. Recent studies have shown that a subset of patients recover from COVID-19 with SARS-CoV-2–specific T cells but not neutralizing antibodies (*6*), indicating a potentially important role for T cells as a mechanism of disease prevention or mitigation. In mouse models, CD8^+^ and CD4^+^ T cells were necessary for protective immunity against SARS and MERS (*28*). Further indication of T cell–mediated disease modification is evident in studies of COVID-19 patient outcomes where lower T cell numbers in patients greater than 60 years of age correlated with increased COVID-19 severity (*7*). Patients who recovered from COVID-19 without requiring intensive care had relatively high T cell levels (*3*–*5*), versus low T cell levels observed in patients who died (*7*) or who had severe disease (*29*), suggesting that an increased level of T cell induction could lead to clinical benefit. Moreover, while antibody responses to SARS-CoV-2, SARS, and MERS are transient and memory B cells are undetectable (*30*–*34*), T cell responses following infection to SARS and MERS endure for decades (*8*, *35*–*37*), indicating that T cells may increase the duration of protection. Several studies have now shown that the natural immune response to SARS-CoV-2 induces CD8^+^ and CD4^+^ T cells specific for epitopes within Spike RBD (*22*), and that these responses persist (*38*).

On the basis of these emerging COVID-19 translational immunogenicity data, in addition to being safe, an optimal SARS-CoV-2 vaccine should (i) induce robust and durable CD8^+^ and CD4^+^ T cell responses, (ii) elicit high-magnitude neutralizing antibodies, (iii) produce T_H_1 bias in the elicited antibody and T cell responses, (iv) potentially expand preexisting cross-reactive T cells, (v) enable dose sparing of required immunogens to improve the speed and cost of broad vaccination campaigns, and (vi) be efficacious in elderly populations.

Here, we describe the evaluation of a lymph node–targeting protein-subunit vaccine, ELI-005, composed of AMP-CpG adjuvant paired with Spike RBD, that exploits the natural trafficking of albumin from subcutaneous tissue at the injection site into lymph nodes to efficiently deliver the adjuvant CpG to antigen-presenting dendritic cells (*15*). Prior studies evaluating CpG as a vaccine adjuvant have demonstrated the importance of achieving lymphatic delivery (*15*, *39*). While conventional unmodified CpG exhibits poor lymphatic accumulation correlated with detectable but modest induction of antigen-specific T cell responses, strategies achieving enhanced delivery of CpG directly into lymph nodes have yielded 10- to 1000-fold stronger T cell responses (*15*). Albumin-binding immunogens and antigens generated using the AMP strategy have proven to be among the most efficient lymph node–targeting agents reported preclinically (*40*). Moreover, albumin-mediated lymphatic transport has been proven effective in humans through extensive use of albumin-binding agents for visualization of sentinel lymph nodes after solid tumor resection (*41*, *42*). Thus, the application of this approach to SARS-CoV-2 holds unique promise to induce potent T cells alongside antibody responses.

Generally, COVID-19 vaccine candidates have induced low T cell responses in mice, with IFNγ^+^ responses of 0 to 2% and 0 to 1% among CD8^+^ and CD4^+^ T cells, respectively (*43*, *44*), or 1000 to 2000 SFCs/1 × 10^6^ splenocytes by IFNγ ELISpot (*19*, *44*, *45*). Consistent with these observations, soluble CpG in combination with Spike-2 protein led to no detectable cytokine production in assays of mouse splenic T cells stimulated with Spike-2 (*46*). In contrast, we observed an increased quantity and enhanced function of T cells responding to ELI-005, inducing 44% of CD8^+^ and 1.5% of CD4^+^ T cells producing IFNγ and TNFα. These T cells were polyfunctional, with a large fraction producing multiple T_H_1 effector cytokines, demonstrating the potential for abundant antigen-presentation and robust costimulation from professional antigen-presenting lymph node cells to drive both expansion and acquisition of potent effector function (*17*). Induced T cells trafficked in large numbers to both lung parenchyma and respiratory secretions, establishing a ready T effector population at a key site of first viral exposure.

Antibody responses to ELI-005 showed 265-fold higher neutralization titers than sera/plasma from convalescent human patients with COVID-19. Although the correlates of protection are not known in mice, ELI-005 exceeded or matched other vaccine candidates in pseudovirus neutralization titers (*19*, *43*–*45*) known to prevent SARS-CoV-2 pneumonia in rhesus macaques (pVNT_50_ 5 to 40) after vaccination with a chimpanzee adenoviral vector vaccine (*20*, *44*) and that led to rapid >3 log_10_ decreases in viral load (pVNT_50_ 74 to 170) after DNA vaccination (*20*).

Previously, the CD4 T_H_2 phenotype has been associated with vaccine-associated enhanced respiratory disease (VAERD) in patients vaccinated against rubella (*47*), respiratory syncytial virus (*48*), and in preclinical studies of potential SARS and MERS vaccines (*49*, *50*). For this reason, we characterized the spectrum of cytokines released from ELI-005–induced T cells in the lung, finding a strong T_H_1 skew with IFNγ and TNFα and no evidence of T_H_2 or T_H_17 cytokine secretion. As with the T cell response, the antibody response showed isotypes associated with T_H_1 skew: IgG2c and IgG3 levels were increased, while IgG1 levels were much lower than comparators. In mice, IgG2a/c isotypes (analogous to human IgG1) are the preferred IgG subclass exhibiting optimal activity against viral infections while IgG1 in mice (and IgG4 in human) are important in allergy, immediate-type hypersensitivity responses, and responses to parasites (*51*). Specifically, murine IgG2a/c exhibits an enhanced ability to induce complement and antibody-dependent cell-mediated cytotoxicity (*52*, *53*). The T_H_1 balance observed in antibody and T cell responses in this study indicates that the potential for T_H_2-associated VAERD in ELI-005–vaccinated patients may be diminished.

Through recent translational studies, several groups (*9*, *22*, *54*) found that patients lacking prior exposure to SARS-CoV-2 have preexisting memory T cell responses that represent cross-reactive T cell clones cognate to common cold coronavirus epitopes. This suggests additional potential benefit for ELI-005 since immunization with AMP-CpG is expected to rapidly expand preexisting pools of cross-reactive T cells resident in the lymph nodes, while other vaccines may have limited ability to activate these memory cells. Since the T cell immune correlates of protection in humans are not yet known for SARS-CoV-2, clinical data correlated to translational T cell immunogenicity results will be required to test this hypothesis.

As there is an urgent need to manufacture an effective vaccine for the entire global community, we examined dose sparing of the Spike RBD protein antigen and evaluated the resulting T cell and antibody responses. Even when the antigen dose was reduced from 10 to 1 μg, AMP-CpG maintained robust T cell responses significantly above alum and soluble CpG. For the humoral response, there was no significant decrease in neutralizing titer as antigen dose decreased from 10 to 1 μg. While dose de-escalation of Spike RBD suggested the antigen dose-sparing potential of ELI-005, the current studies did not evaluate reduced doses admixed with comparator adjuvants. Nonetheless, these results indicate that ELI-005 may have a reduced requirement for antigen dose, which could speed the delivery of protein subunit vaccines to large populations and reduce the cost and logistical burden of manufacturing efforts. Immunization with AMP-CpG elicited similar immunity following either two or three dose regimens, indicating the potential for rapid induction of responses with limited dosing. There is further potential for a stable liquid ELI-005 vaccine formulation, simplifying distribution without frozen shipment as AMP-CpG maintains activity when stored at 4°C for >14 days.

Effective immunization in the elderly is challenging because of immunological senescence. Soluble CpG is used as the adjuvant for the marketed hepatitis B vaccine, Heplisav-B; with this preparation, the proportion of 60- to 70-year-olds who achieved seroprotection was 27.3% higher than that of alum (*55*), suggesting that agonism of the toll-like receptor 9 (TLR-9) pathway via CpG improves responses in this age group. Therefore, we investigated the potential for ELI-005 to elicit immune responses in aged mice. While neutralizing antibody titer and peripheral blood T cell responses were reduced compared to younger mice, they were still significantly stronger than those induced by soluble CpG or alum comparators, and in excess of levels observed in convalescent patients. Encouragingly, elevated AMP-CpG induced T cell and antibody responses were maintained 6 weeks after immunization, indicating that memory T cell responses were induced.

While the current data assessed responses to SARS-CoV-2, the general features of the induced T cell response recapitulated prior observations of AMP vaccination (*16*) in oncology, while demonstrating parallel potential to stimulate neutralizing antibodies, suggesting broad potential for generating target agnostic, potent, and balanced immune responses. Further translational research will be needed to assess the prophylactic capacity and durability of the responses to SARS-CoV-2. Despite distinct TLR-9 expression patterns between murine and human innate immune cells, TLR-9 agonism using B-type CpG oligonucleotides in humans has proven effective to induce cellular and humoral immunity in prior clinical studies (*55*, *56*), suggesting potential for effective translation of ELI-005 to human use. Furthermore, modification of alternative CpG subclasses with distinct mechanisms for activation through TLR-9 presents an important opportunity for generation of AMP adjuvants through enhanced lymphatic targeting. While subcutaneous dosing was used in the current studies, mechanisms of albumin biodistribution from diverse interstitial sites are conserved, presenting the opportunity to evaluate alternative dosing routes to facilitate simple widespread administration.

AMP-CpG has the potential to enhance a broad spectrum of vaccines. The promising immunogenicity results reported here for ELI-005 support its clinical evaluation as a candidate vaccine for SARS-CoV-2.

## MATERIALS AND METHODS

### Vaccine components

Vaccines consisted of SARS-CoV-2 Spike-2 RBD protein (Genscript; Cat: Z03483), combined with alhydrogel (InvivoGen, catalog no. vac-alu-250), soluble CpG 1826 (InvivoGen, catalog no. TLRL-1826), or AMP-CpG 1826 as indicated. Alternatively, soluble CpG 7909 (InvivoGen, catalog no. TLRL-2006) or AMP-CpG 7909 was used where indicated. Adjuvant-only treatment groups received a matching dose of adjuvant in the absence of antigen. Mock treatment groups were treated with phosphate-buffered saline (PBS) alone.

### Animals

Female, 6- to 8-week-old C57BL/6J and BALB/c mice and 37-week-old C57BL/6J mice were purchased from the Jackson Laboratory (Bar Harbor, ME). All animal studies were carried out under an institute-approved Institutional Animal Care and Use Committee (IACUC) protocol following federal, state, and local guidelines for the care and use of animals. Mice were injected with the indicated concentrations of Spike RBD protein, and soluble CpG (CpG), lipid-conjugated CpG (AMP-CpG), or alum admixed with PBS. Injections (100 μl) were administered subcutaneously at the base of the tail (50 μl bilaterally) on days 0, 14, and 28. Blood samples were collected on days 7, 21, and 35. Mice were euthanized on day 35 for collection of BAL fluid followed by lung harvest in the same animals. Only the pilot experiment (Fig. 1) received a fourth dose on day 42, and samples were collected on day 49. BAL fluid was obtained by washing the lungs three times each with 1 ml of PBS. Lungs were harvested following perfusion with 10 ml of PBS into the right ventricle of the heart. Lung tissue was physically dissociated and digested with RMPI 1640 media containing collagenase D (1 mg/ml) and deoxyribonuclease I (25 U/ml).

### Human convalescent serum/plasma

Convalescent serum samples (*n* = 7) and plasma samples (*n* = 15) from patients who had recovered from SARS-CoV-2 infection (COVID-19) were obtained from US Biolab (Rockville, MD) and AllCells (Alameda, CA), respectively. All samples were received and stored frozen at −80°C until analysis.

### Antigen-binding ELISA

ELISAs were performed to determine sera antibody binding titers. ELISA plates were coated with CoV-2 RBD protein (200 ng per well; GenScript; catalog no. Z03483) overnight at 4°C. Plates were preblocked with 2% bovine serum albumin for 2 hours at room temperature (RT). Serially diluted mouse sera were transferred to the ELISA plates and incubated for 2 hours at RT. Plates were washed four times with washing buffer (BioLegend; catalog no. 4211601) and then incubated for 1 hour at RT with a 1:2000 dilution of secondary antibody of horseradish peroxidase (HRP)–conjugated rabbit anti-mouse IgM (μ chain), HRP-rabbit anti-mouse IgG (Fcγ), HRP-goat anti-mouse IgG1, HRP-goat anti-mouse IgG2c, or HRP-goat anti-mouse IgG3 (Jackson ImmunoResearch, catalog nos. 315-035-048, 315-035-049, 315-035-046, 115-035-205, 115-035-207, 115-035-208, and 115-035-209, respectively). Plates were again washed four times with washing buffer, after which the plates were developed with 3,3′,5,5′-tetramethytlbenzidine for 10 min at RT, and the reaction was stopped with 1 N sulfuric acid. The absorbance at 450 nm was measured by an ELISA plate reader. Titers were determined at an absorbance cutoff of 0.5 OD (optical density).

### SARS-CoV-2 pseudovirus neutralization assay

For the neutralization assay, we used the ACE2–human embryonic kidney–293 (HEK293) recombinant cell line (BPS Bioscience, catalog no. 79951) or the control HEK293 cell line (American Type Culture Collection) and the SARS-CoV-2 Spike Pseudotyped Lentivirus (BPS Bioscience, catalog no. 79942). The SARS-CoV-2 Spike Pseudotyped Lentivirus contains the luciferase reporter gene and the SARS-CoV-2 Spike envelope glycoproteins, thus specifically transducing ACE2-expressing cells. Mouse or human sera dilutions were performed in the Thaw Medium 1 (BPS Bioscience, catalog no. 60187) in 96-well white clear-bottom luminescence plates (Corning, catalog no. 3610) and then preincubated with 10 μl of virus for 30 min at RT. ACE2-HEK293 or control HEK293 cells (40 μl), containing 10,000 cells, were then added to the wells and incubated at 37°C for 48 hours. Control wells included ACE2-HEK293 cells or control HEK293 cells with the virus, but no sera, and provided the maximum transduction level and the background, respectively. Luciferase activity was detected by adding 70 μl of freshly prepared ONE-Step Luciferase reagent (BPS Bioscience, catalog no. 60690) for 15 min at RT and luminescence was measured with a Synergy H1 Hybrid reader (BioTek). Pseudovirus neutralization data for the pilot experiment was performed by GenScript (Nanjing, China) following the same protocol, but using in-house ACE2-HEK293 cells and Spike RBD-HRP recombinant protein. Pseudovirus neutralization titers at pVNT_50_ were calculated as the serum dilution at which relative luminescence units (RLU) were reduced by 50% compared to RLU in virus control wells.

### ACE2 competition ELISA

For the ACE2 competition ELISA, the cPass kit from GenScript was used (catalog no. L00847). Manufacturer’s instructions were followed. In short, serially diluted sera were incubated with SARS-CoV-2 Spike RBD-HRP and added to ACE2 precoated plates. Plates were developed with 3,3′,5,5′-tetramethytlbenzidine for 10 min at RT, and the reaction was stopped with 1 N sulfuric acid. The absorbance at 450 nm was measured by an ELISA plate reader. Titers were determined at an absorbance cutoff of 0.5 OD.

### Lung T cell cytokine determination by CBA

Cytometric bead array (CBA) flow cytometry was performed to determine cytokine production. Lung leukocytes (collected after the final booster dose) were activated overnight with SARS-CoV-2 Spike glycoprotein overlapping peptides at 1 μg per peptide per well [consisting of 315 peptides, derived from a peptide scan (15-mers with 11 amino acid overlap) through Spike glycoprotein of SARS-CoV-2] (JPT, catalog no. PM-WCPV-5 or GenScript, catalog no. RP30020). Phorbol myristate acetate (PMA; 50 ng/ml) and ionomycin (1 μM) were used as positive controls, and complete medium only was used as the negative control. Culture supernatants were harvested, and T_H_1/T_H_2 cytokine production was measured (CBA Mouse Th1/Th2/Th17 Cytokine Kit: BD, catalog no. BDB560485). Briefly, bead populations with distinct fluorescence intensities that are coated with capture antibodies specific for various cytokines including IFNγ, TNFα, IL-4, IL-6, IL-10, and IL-17 were incubated with culture supernatants. The different cytokines in the sample were captured by their corresponding beads. The cytokine-captured beads were then mixed with phycoerythrin (PE)–conjugated detection antibodies. Following incubation, samples were washed, and fluorescent intensity of PE on the beads were measured and analyzed by flow cytometry (BD FACSCanto II). Mean fluorescent intensities were calculated using FACSDiva V8.0.1 software (BD) and protein concentrations were extrapolated using Microsoft Excel.

### Peripheral blood and lung T cell cytokine determination by ICS

Intracellular cytokine staining (ICS) was performed for TNFα and IFNγ. Peripheral blood cells (collected 7 days after each booster dose) and lung leukocytes (collected after the final booster dose) were stimulated overnight with 1 μg per peptide per well of Spike-derived overlapping peptides at 37°C, 5% CO_2_ in the presence of brefeldin A (Invitrogen, catalog no. 00-4506-15) and monensin (BioLegend, catalog no. 420701). Cells were stained with the following antibodies: PE anti-mouse IFNγ (BD, catalog no. 554412), fluorescein isothiocyanate anti-mouse TNFα (BD, catalog no. 554418), APC-Cy7 anti-mouse CD3 (BD, catalog no. 560590), PE-Cy7 anti-mouse CD4^+^ (Invitrogen, catalog no. 25-0041-82), and APC anti-mouse CD8a (eBioscience, catalog no. 17-0081-83). PMA (50 ng/ml) and ionomycin (1 μM) were used as positive controls, and complete medium only was used as the negative control. Cells were permeabilized and fixed (Invitrogen, catalog no. 00-5523-00). A LIVE/DEAD fixable (aqua) dead cell stain kit (Invitrogen, catalog no. L34966) was used to evaluate viability of the cells during flow cytometry. Sample acquisition was performed on FACSCanto II (BD) and data were analyzed with FlowJo V10 software (BD).

### IFNγ ELISpot

Spleens from mice were collected individually in RPMI 1640 media supplemented with 10% fetal bovine serum and penicillin, streptomycin, nonessential amino acids, sodium pyruvate, and β-mercaptoethanol (complete media) and then processed into single-cell suspensions and passed through a 70-μm nylon filter. Cell pellets were re-suspended in 3 ml of ACK lysis buffer (Quality Biological Inc., catalog no. 118156101) for 5 min on ice; then, PBS was added to stop the reaction. The samples were centrifuged at 400*g* for 5 min at 4°C, and cell pellets were re-suspended in complete media. ELISpot assays were performed using the Mouse IFNγ ELISpot Set (BD, catalog no. BD551083). Ninety-six–well ELISpot plates precoated with capture antibody overnight at 4°C were blocked with complete media for 2 hours at RT. Mouse splenocytes (500,000) were plated into each well and stimulated overnight with 1 μg per peptide per well of Spike-derived overlapping peptides. The spots were developed on the basis of the manufacturer’s instructions. PMA (50 ng/ml) and ionomycin (1 μM) were used as positive controls, and complete medium only was used as the negative control. Spots were scanned and quantified by an ImmunoSpot CTL reader.

### Statistics

All data were plotted and all statistical analyses were performed using GraphPad Prism 8 software (La Jolla, CA). All graphs display mean values, and the error bars represent the standard deviation. No samples or animals were excluded from the analyses. Animals were not randomized for any of the studies, and dosing was not blinded. Statistical comparisons between groups were conducted using two-sided Mann-Whitney tests. Data were considered statistically significant if the *P* value was less than 0.05.

## SUPPLEMENTARY MATERIALS

Supplementary material for this article is available at http://advances.sciencemag.org/cgi/content/full/7/6/eabe5819/DC1

## Appendix

View/request a protocol for this paper from *Bio-protocol*.
